# Insights into short‐ and long‐term crop‐foraging strategies in a chacma baboon (*Papio ursinus*) from GPS and accelerometer data

**DOI:** 10.1002/ece3.7114

**Published:** 2021-01-03

**Authors:** Ben J. Walton, Leah J. Findlay, Russell A. Hill

**Affiliations:** ^1^ Department of Anthropology University of Durham Durham UK; ^2^ Primate & Predator Project Lajuma Research Centre Louis Trichardt (Makhado) South Africa; ^3^ Department of Zoology University of Venda Thohoyandou South Africa

**Keywords:** bio‐logging, crop‐raiding, human–wildlife conflict, human–wildlife interactions

## Abstract

Crop‐foraging by animals is a leading cause of human–wildlife “conflict” globally, affecting farmers and resulting in the death of many animals in retaliation, including primates. Despite significant research into crop‐foraging by primates, relatively little is understood about the behavior and movements of primates in and around crop fields, largely due to the limitations of traditional observational methods. Crop‐foraging by primates in large‐scale agriculture has also received little attention. We used GPS and accelerometer bio‐loggers, along with environmental data, to gain an understanding of the spatial and temporal patterns of activity for a female in a crop‐foraging baboon group in and around commercial farms in South Africa over one year. Crop fields were avoided for most of the year, suggesting that fields are perceived as a high‐risk habitat. When field visits did occur, this was generally when plant primary productivity was low, suggesting that crops were a “fallback food”. All recorded field visits were at or before 15:00. Activity was significantly higher in crop fields than in the landscape in general, evidence that crop‐foraging is an energetically costly strategy and that fields are perceived as a risky habitat. In contrast, activity was significantly lower within 100 m of the field edge than in the rest of the landscape, suggesting that baboons wait near the field edge to assess risks before crop‐foraging. Together, this understanding of the spatiotemporal dynamics of crop‐foraging can help to inform crop protection strategies and reduce conflict between humans and baboons in South Africa.

## Graphical abstract

A GPS and accelerometer collar was deployed to understand crop‐foraging by chacma baboons in South Africa. This gave insights into the spatiotemporal patterns of crop‐foraging baboon behavior and indicated that crop‐foraging occurred when natural food availability was low and that baboons waited on the edge of crop fields before engaging in high activity forays to access crops. We discuss how these behavioral insights can inform crop protection strategies to reduce human–baboon conflict.

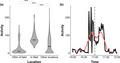

## 1 INTRODUCTION

Agroecosystems now cover more than one quarter of global land area (Altieri & Koohafkan, 2004), which combined with widespread habitat degradation has resulted in many species incorporating anthropogenic food sources into their diets (Hill, 2018). Primates form a large proportion of the literature on crop‐foraging (Hill, 2018). Their capacity for learning and behavioral flexibility (McLennan et al., 2017) mean that they can overcome many crop protection strategies and dietary flexibility in many primates (Chapman, 1987; McLennan et al., 2017) allows them to exploit a wide range of crop types. Many primates travel in large social groups and are likely to receive attention from farmers (Hill, 2000) and can have significant socioeconomic impacts on rural farming communities (Haule et al., 2002; Hill & Wallace, 2012; Kagoro‐Rugunda, 2004; Mwakatobe et al., 2014; Nyhus et al., 2005). Lethal control is a common response to crop‐foraging by primates (Findlay, 2016; McLennan et al., 2012; Pahad, 2010), and with 60% of primate species now threatened with extinction (Estrada et al., 2018), understanding conflict between humans and primates is increasingly important for primate conservation globally, as well as for local economies and food security.

To properly address the issues that arise because of crop‐foraging by primates, it is useful to understand the ecological drivers of crop‐foraging, as this understanding can in turn inform the timing and implementation of crop protection strategies. In many primate species, crop‐foraging varies on a seasonal basis, often increasing when natural food availability is low (De Freitas et al., 2008; Findlay, 2016; Hockings et al., 2009; Naughton‐Treves et al., 1998; Pahad, 2010; Strum, 2010), with crops potentially acting as a “fallback food” (Hill, 2018; Lambert & Rothman, 2015; Marshall & Wrangham, 2007). Behavioral observations and interviews with farmers suggest that there is a seasonal pattern to crop‐foraging by chacma baboons on commercial farms in South Africa, with greater impacts in the dry season when plant productivity is low (Findlay, 2016). Crop‐foraging may also vary depending on the seasonal availability of crop foods (Seiler & Robbins, 2016). However, the benefits of feeding on anthropogenic foods can be numerous (Chiyo et al., 2011; Higham et al., 2009; Strum, 2010) and crop‐foraging may not follow any seasonal pattern if the benefits outweigh the risks throughout the year.

Crop‐foraging by animals may also vary on a shorter temporal scale; Buton macaques (*Macaca ochreata brunnescens*) in Sulawesi crop‐foraged more frequently in the morning (Priston, 2005), whereas primates in Uganda foraged on crops between noon and sunset more than between sunrise and noon (Wallace, 2010). In a study of a baboon group foraging on commercial farms in South Africa, baboons were more likely to forage in the morning than the afternoon (Findlay & Hill, In press). It was suggested that for these baboons, greater crop‐foraging in the morning may be explained by the close proximity of sleeping sites to the fields. These differences in the diurnal pattern of crop‐foraging are likely to be site‐ and group‐dependent so may not be applicable beyond a local context but can still inform the timing of protection strategies at a local level.

Crop protection strategies can also be informed by an understanding of the behavior of crop‐foraging animals, as animals may employ a number of behavioral “strategies” to minimize the risks of entering fields. For example, chimpanzees (*Pan troglodytes*) have been shown to forage in crops at night (Krief et al., 2014) and reduce vocalization rates in crop fields (Wilson et al., 2007). Chacma baboons (*Papio ursinus*) used a sit‐and‐wait strategy—high activity foraging forays into anthropogenic areas combined with periods of low activity on the edge of urban areas, likely assessing risk—to minimize risks and maximize rewards associated with foraging in an anthropogenic habitat (Fehlmann, O'Riain, Kerr‐Smith, et al., 2017). Similar high activity forays have also been observed on commercial farms in South Africa, where baboons run into fields to collect crops and then consume them outside of fields (Findlay, 2016).

However, despite significant research, our understanding of the spatiotemporal patterns of primate crop‐foraging remains relatively poor. Where crop‐foraging patterns have been recorded, this is generally within the confines of a small number of fields, across limited times within days and weeks and only within or just outside of fields (e.g., Findlay & Hill, In press; Wallace & Hill, 2012). This is largely because traditional methods of primate observation rely on habituation, which poses significant ethical and feasibility issues in this context. Habituation may not be possible with animals that are regularly chased and threatened by humans and is also unlikely to be ethical given the possible link between habituation and increased crop‐foraging (Fedigan, 2010; Green & Gabriel, 2020; Madden, 2006; Seiler & Robbins, 2016). Therefore, there is a need to find an alternative way to understand what primates do both in and out of crop fields and to understand their movement patterns in greater detail than is possible by direct observation.

On‐animal technology, or bio‐logging (Boyd et al., 2004; Fehlmann & King, 2016), has the potential to overcome these limitations. GPS can give information on the location of animals, and accelerometers can provide data on their activity patterns. Combined, these can answer questions that observational methods cannot. Though GPS collars have been used previously to try to gain insights into primate crop‐foraging, this has been at a low resolution (one GPS fix every three hours, Pahad, 2010), which limits the conclusions that can be drawn. Data at a higher temporal resolution can allow better identification of the timing of specific crop‐foraging events. Accelerometers have not been used previously to understand baboon crop‐foraging, though combined GPS and accelerometers have been used to understand baboon foraging in an urban setting in Cape Town, South Africa (Fehlmann, O'Riain, Kerr‐Smith, et al., 2017).

Remote sensing of ecological variables can be combined with data from bio‐loggers to understand the ecological drivers of baboon behavior. The Normalised Difference Vegetation Index (NDVI) is a commonly used index of both primary productivity and vegetation structure (Myneni et al., 1995) and therefore gives an indication of vegetation availability. While NDVI is not a direct measure of primate food availability, it can be used with caution to make inferences about broader food availability on a coarse temporal scale. Sensible deductions can be made about fruit and seed availability using NDVI, as these are likely to mature after seasonal peaks in primary productivity. Willems et al., 2009 found NDVI to be quadratically associated with vervet food availability across the year, and it also related to patterns of vervet monkey movement. In a study of food availability for Japanese macaques (*Macaca fuscata*), Tsuji et al. (2015) found a linear relationship between NDVI and seed and fruit availability, the preferred food of baboons (Hill & Dunbar, 2002). It is also likely a good measure of leaf and grass availability, which do constitute an important part of the baboon diet (Hill & Dunbar, 2002) in many populations.

Baboons (*Papio* spp.) in southern Africa are often cited by farmers as the vertebrate taxa that cause the greatest crop loss, and they are regularly shot or killed by farmers and chased by field guards (Findlay, 2016; Hill, 2000; Mwakatobe et al., 2014). While there is a body of literature on baboon crop‐foraging in subsistence agriculture (Hill, 2018), there is a lack of understanding of the patterns of baboon crop‐foraging in a commercial context. Commercial farms are much larger and individual farmers are often better resourced to trap and kill baboons. These differences mean that effective management strategies are also likely to be different. Previous research in our study area has not been able to describe baboon activity quantitatively and has not addressed what baboons do on the edge of fields or in the broader agricultural landscape (Findlay & Hill, In press; Findlay, 2016). Therefore, gaining a better understanding of the behavior of crop‐foraging baboons in this context is a priority for addressing human–wildlife conflict in the region.

In this paper, we use GPS and accelerometer data from a collar deployed on a baboon in the Limpopo Province of South Africa to explore the behavior and space use of crop‐foraging baboons. Although data from only one collar were recovered, this still gives an insight into the patterns of movement and activity of the whole group, as baboon groups tend to move as a cohesive unit (Sueur, 2011). Previous research in the area has shown that baboons often raid as a group, with up to 63 baboons recorded in a single field visit (Findlay, 2016). GPS data from a single collar have also given useful insights into baboon movement and ecology in the past (Pebsworth et al., 2012).

Based on previous research (Fehlmann, O'Riain, Kerr‐Smith, et al., 2017; Findlay & Hill, In press; Findlay, 2016), it was predicted that the baboon would crop forage strategically due to the risks and rewards associated with crop‐foraging. Specifically, (a) the baboon would generally avoid fields as they are perceived as high risk; (b) field visits would occur when natural food was low and crops were present, as crops are a fallback food; (c) crop‐foraging would be greater in the morning than the afternoon, based on research on another baboon group in the study region; (d) activity in fields would be high because the baboon would use high‐intensity foraging to minimize risk of detection and harm; and (e) activity on the field periphery would be low because the baboon would engage in vigilance and low activity behaviors to assess risk, determine the location of the field guards, and assess foraging opportunities in the field.

## 2 MATERIALS AND METHODS

### 2.1 Study location

Fieldwork was conducted in South Africa in the Blouberg District Municipality, in the northernmost part of the Limpopo Province, South Africa. The climate of the study area is semiarid, with warm summers (October–March) and cooler, dryer winters (April–September). Most rainfall occurs in the months of summer and drought is common in the area. The vegetation of the study area is savanna, locally known as “bushveld,” which provides food for baboons when not feeding on crops, in the form of fruits, tubers, seeds, leaves, and some animal matter (Hamilton et al., 1978). This habitat runs up to the edges of crop fields. Leopards (*Panthera pardus*) are the most significant predator of baboons in the study area, though numbers are thought to be low (Jamie McKaughan, unpublished data). There are also several watercourses in the area, tributaries to the Mogalakwena River, providing water for field irrigation and supporting trees and larger vegetation that provide sleeping sites for baboons.

Agriculture is economically significant for the region, with both large‐scale commercial farms and smaller subsistence farms, with commercial farms generally owned by white Afrikaans‐speaking farmers. Common crops include tomatoes, potatoes, pumpkins, squashes, and tobacco. The study fields were all part of a large‐scale commercial farm. A small number of buildings, along with some fences, tracks, and hides, were located at the site, although the human presence was still low in the study area. Chacma baboons are regularly cited by local farmers as the vertebrate species responsible for greatest crop losses (Findlay, 2016) and farmers hire crop guards to chase baboons when crops are present and also occasionally shoot baboons when in or near to crop fields.

### 2.2 Baboon collaring and collar recovery

Two unhabituated chacma baboon groups known to forage on crops were chosen for collaring. One adult female from each group was fitted with a GPS and accelerometer collar with an automatic timer‐activated drop‐off (Vectronic GPS‐PLUS collars 18; VECTRONIC Aerospace). Collars weighed less than 5% of the body mass of the baboons. Due to the presumed failure of the VHF transmitter on one collar, only a single collar was recovered for data analysis. The recovered collar was on a female baboon in a group of 44 individuals. All procedures were approved by the University of Durham Animal Welfare Ethical Review Board and Limpopo Provincial Government Department of Economic Development, Environment and Tourism.

A single 2 × 0.8 × 0.9 m cage trap baited with butternut squash was used to capture the baboons. It was opened in the morning and checked regularly. When an adult female weighing over 10 kg was caught, the cage was covered with tarpaulin to calm the animal down and the vet was called immediately. Baboons were sedated with a combination of 0.03 mg/kg medetomidine hydrochloride (Domitor; Pfizer Laboratories (Pty) Ltd) and 50 mg ketamine delivered by pole syringe. Animals were then fitted with collars. Baboons were weighed by the vet during the procedure. The medetomidine hydrochloride was then antagonized with an intramuscular dose of atipamezole (Antisedan; Pfizer Laboratories (Pty)), given at 1 mg, and animals were kept under observation while recovering in the crate before being released to rejoin their group. Animal behavior postrelease was also observed.

### 2.3 Collar programming and analysis

The collar recorded data for full days between 26/10/2013 and 18/10/2014. The collar took hourly GPS fixes from 05:00 to 19:00 South African Standard Time, with a further nocturnal fix at 24:00 to identify sleeping sites (*N* = 5,728 fixes). There were no unsuccessful GPS fixes and the mean time to a GPS fix was 28 s, as the study area is flat and has sparse vegetation so device to satellite communication was unimpeded. The collar also contained a biaxial accelerometer, positioned at the back of the baboon's neck, which recorded acceleration in the *x*‐axis and *y*‐axis at 4 Hz. The *x*‐axis accelerometer recorded movement from front to back (surge) and the *y*‐axis accelerometer from side to side (sway). The collar averaged acceleration in the two axes over 120 s for storage. Activity values range from 0 (fully at rest) to 255. The GPS collar was fitted with an automatic drop‐off, which fell off approximately 365 days after the collars were fitted. UHF was periodically used to download data from the collar and VHF was used to retrieve the collar once it dropped off the baboon.

There was a strong positive correlation between acceleration in the two axes (Pearson's correlation coefficient, *r* = .96, *p* = <.0001); therefore, only acceleration data for the *x*‐axis were analyzed (Ayers et al., 2019; Heurich et al., 2014). Activity at any given GPS point was taken as the average of the acceleration in the *x*‐axis over the two minutes preceding and two minutes  following the GPS fix. Where duplicate values existed for a particular time because of errors in data storage, the first value for acceleration was used. Where a value was missing, a single value of acceleration was used for the relevant four‐minute time window.

### 2.4 Ecological variables

Normalised Difference Vegetation Index (NDVI) was used as a measure of primary productivity across the home range. NDVI is a dimensionless index between −1 and 1, where values closer to 1 indicate greater plant primary productivity. Data from the Moderate Resolution Imaging Spectrometer (MODIS) on board of NASA's TERRA satellite were retrieved using the Google Earth Engine website (https://earthengine.google.com, Gorelick et al., 2017), and monthly NDVI values were downloaded from the MOD13Q1 data set (250‐m spatial resolution) over the home range of the study group (based on a minimum convex polygon of 62.7 km^2^). Pixels were discarded based on associated quality assessment data sets, where only pixels with associated quality values of 0 (good) were retained. Average NDVI values were then taken from the remaining pixels for each month.

### 2.5 Statistical tests and data analysis

Daily path length was calculated by measuring the straight‐line distances between hourly GPS fixes over the course of one day (midnight to midnight). A linear mixed model was used to assess the relationship between NDVI and daily path length, with month included as a random effect. A Kenward–Roger *F*‐test was used to assess whether the relationship was statistically significant.

Home range was calculated using kernel density estimates using the R package adehabitat R (Calenge, 2006). Although the “least squares cross‐validation” (LSCV) method is often cited as the best method for estimating the bandwidth (or smoothing parameter) for kernel density estimates (e.g., Erran Seaman & Powell, 1996), in this case the LSCV method failed to estimate the bandwidth (*h*), probably because of repeated sampling in the same location at sleeping sites (Seaman et al., 1998). Similar problems have been found in other instances of wildlife tracking where the study species has high site fidelity (Hemson et al., 2005). Therefore, the reference bandwidth (*h*_ref_) was used to calculate 95%, 90%, and 50% isopleths. Home range was based on 95% kernel density estimates and core home range was based on 50% kernel density estimates.

To test for differences in activity by location, we compared activity for two minutes before and after GPS fixes in different areas, excluding nocturnal fixes (at 24:00). We compared activity associated with fixes in fields to activity at fixes within 100 m of field edges and compared both with activity for locations beyond 100 m of field edges. 100 m was chosen as an appropriate buffer to investigate the behavior of baboons just outside the fields as previous research suggested that baboons spend a significant amount of time in this area prior to entering fields (Findlay, 2016). Nonparametric tests were used as diagnostic plots showed that the activity data were not normally distributed due to a high number of zero values; a Kruskal–Wallis rank‐sum test was used to compare all locations, followed by a pairwise Wilcoxon rank‐sum test with *p*‐values adjusted for multiple comparisons (Benjamini & Hochberg, 1995). Statistical and spatial analyses were conducted in R version 3.6.1 with α set at 0.05 (R Core Team, 2020) and ArcGIS Pro version 2.4.2.

## 3 RESULTS

### 3.1 Yearly ranging patterns

**FIGURE 1:**
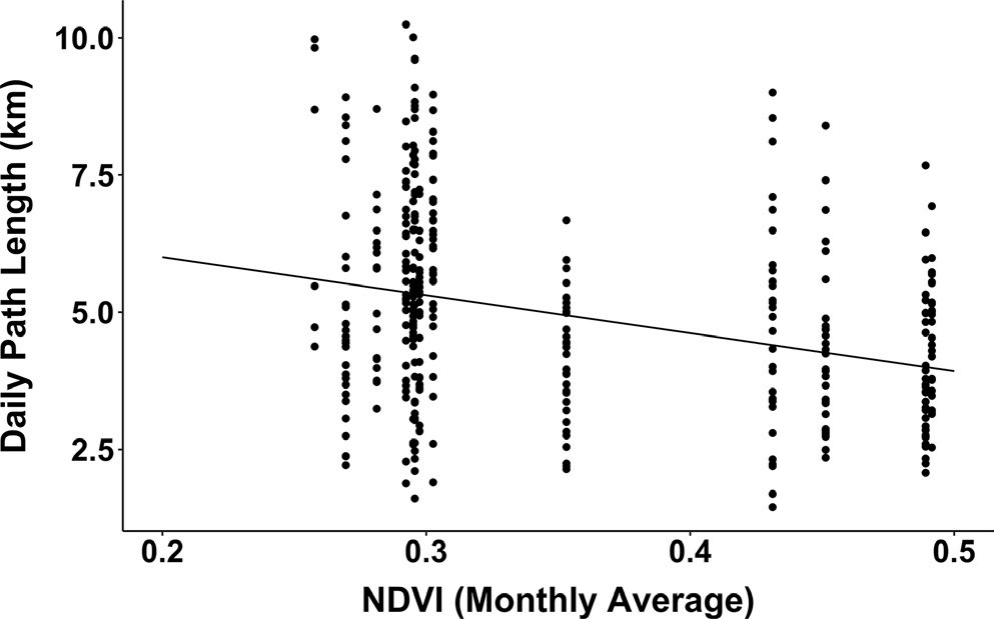
Relationship between daily path length and Normalised Difference Vegetation Index (NDVI)

Average daily path length was 4.99 km, with a minimum path length of 1.45 km and a maximum of 10.24 km, though these distances are an underestimation of actual daily distance traveled given the sampling interval. Daily path length decreased as monthly NDVI increased (Figure 1, linear mixed model: NDVI: estimate (±*SE*) = −6.92 ± 1.91, Kenward–Roger *F*‐test: *F*_1,11_ = 13.0, *p* = .004).

**FIGURE 2:**
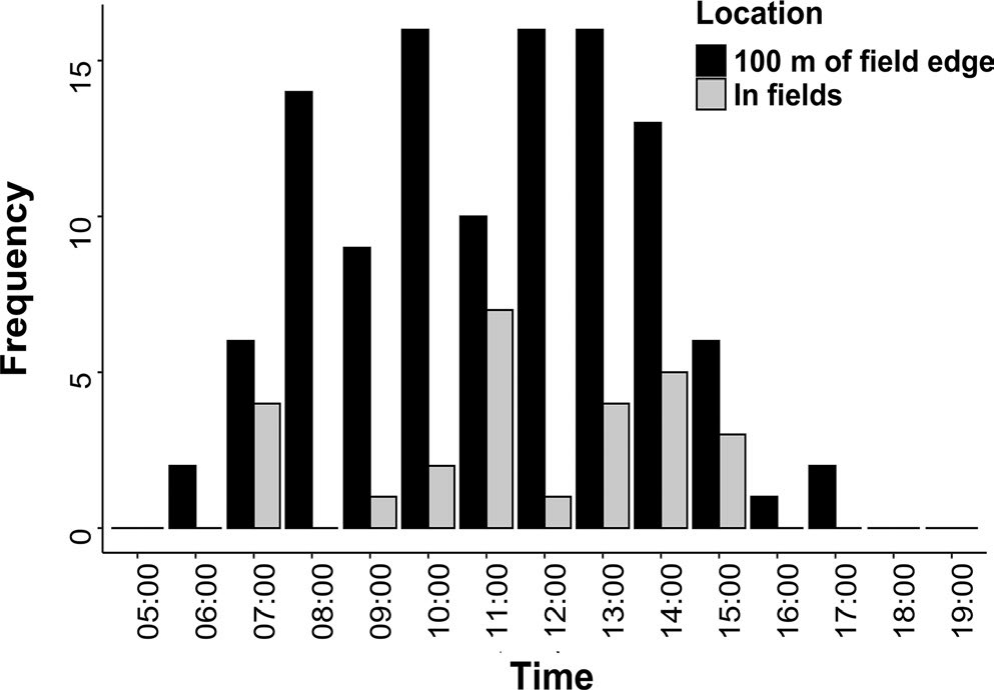
Time of day when the baboon was in the field or within 100 m of the field edge

**FIGURE 3:**
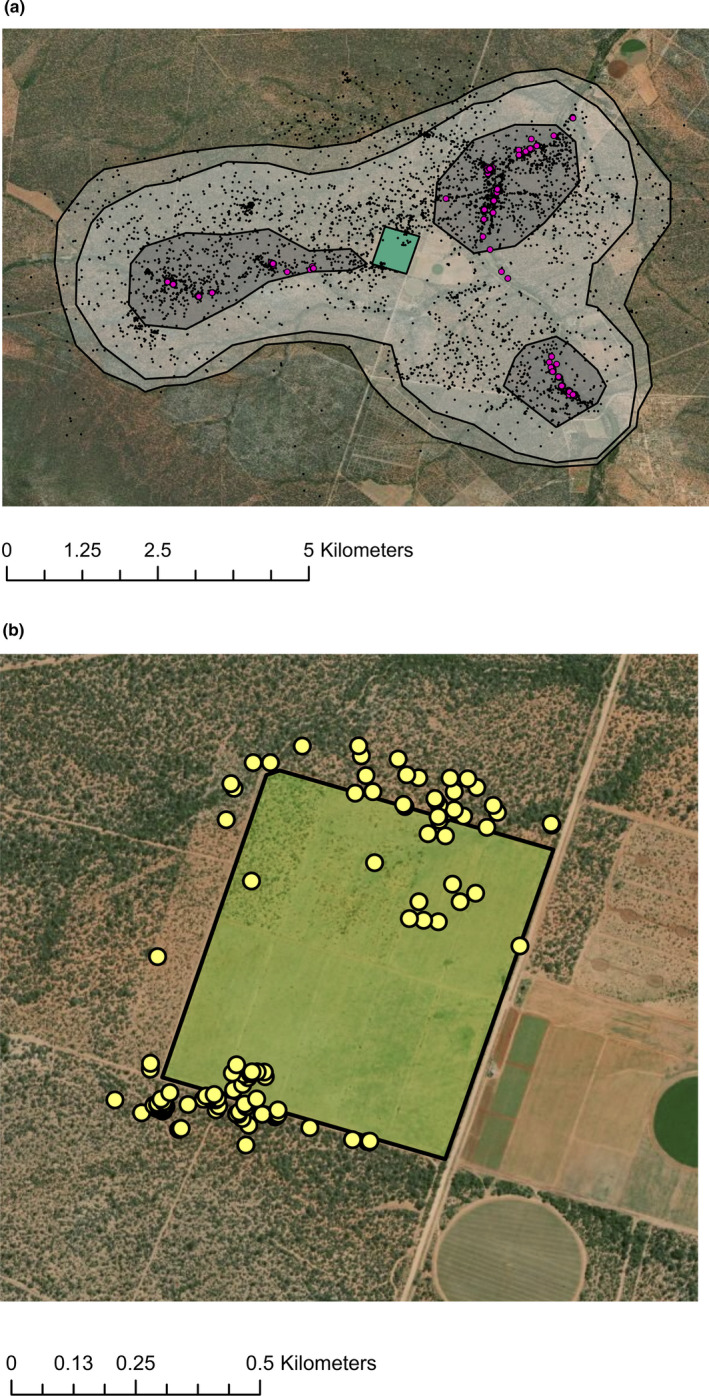
(a) GPS points recorded for the study baboon over approximately one year (black) with sleeping sites (pink). 95%, 90%, and 50% home range isopleths based on kernel density estimates (gray). The position of the fields relative to the GPS points (green). (b) GPS points within the fields and within 100 m of the fields (yellow). Other fields visible on map were not present at the time of data collection

**FIGURE 4:**
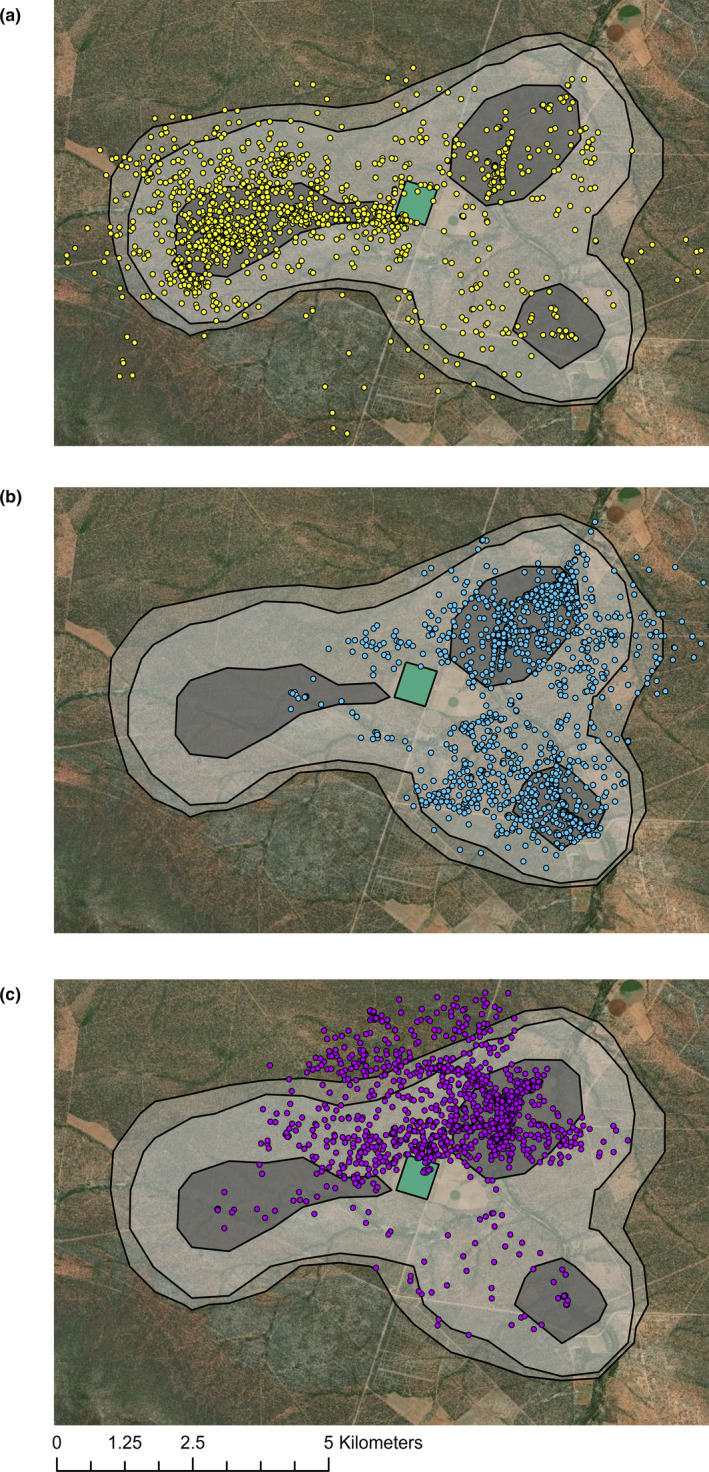
Changes in baboon ranging patterns through the year, with 95%, 90%, and 50% home range isopleths based on kernel density estimates (gray). (a) October 2013–February 2014, (b) March 2014–June 2014, (c) July 2014–October 2014

The study baboon was recorded in the fields 27 times across 24 days and was recorded within a 100 m buffer of the field 111 times in total. No field visits were recorded by the GPS after 15:00 and only 2.7% of fixes within 100 m of the field edge occur after 15:00 (Figure 2). Ninety‐five percent home range was 40.8 km^2^ with a core home range of 8.21 km^2^. The core home range formed three separate areas and did not overlap with the fields (Figure 3). Spatial distribution of the baboon ranging patterns changed throughout the year (Figure 4).

### 3.2 Environmental predictors of crop‐foraging

**FIGURE 5:**
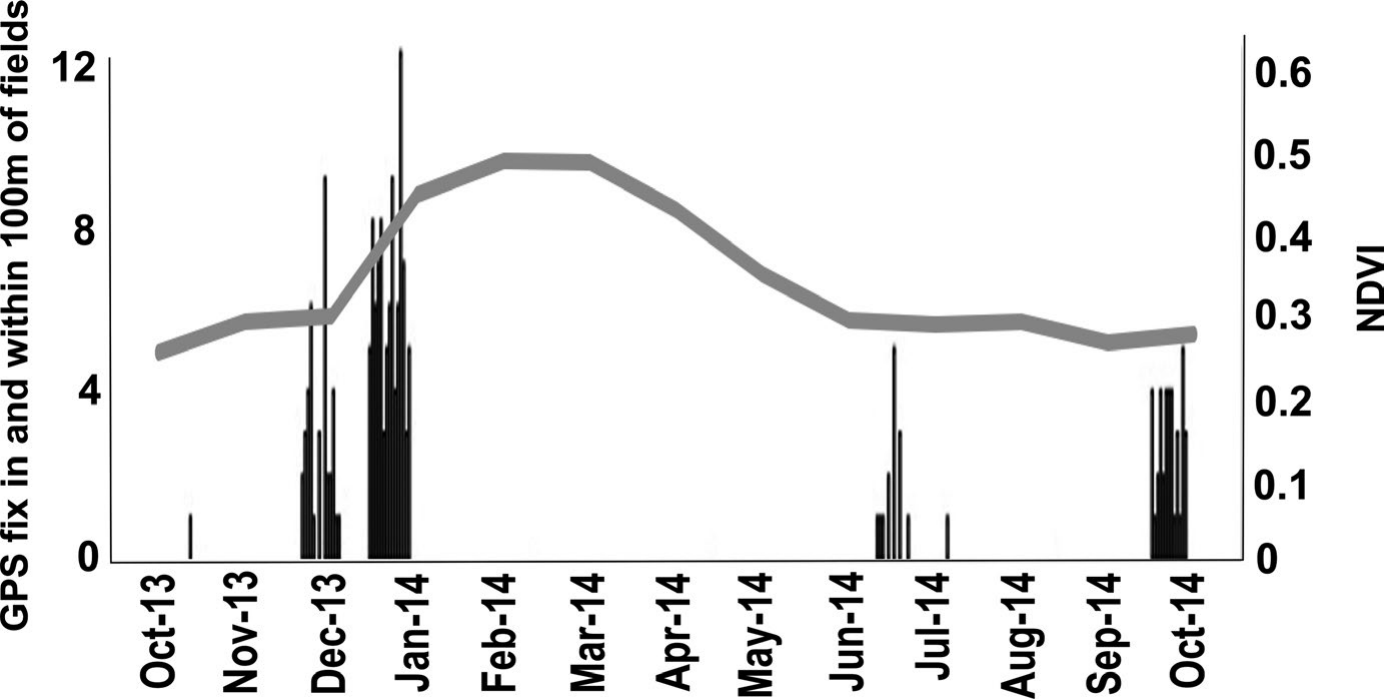
Mean monthly Normalised Difference Vegetation Index (NDVI; gray line) and number of GPS fixes for each day in fields or within 100 m of fields (black bars)

The study baboon was recorded in the fields and within a 100 m buffer of the fields in October 2013, December 2013, January 2014, June 2014, July 2014, and October 2014. These GPS fixes were generally temporally clustered on consecutive days. With the exception of January, these times were when mean NDVI across the home range was low (<0.31; Figure 5).

### 3.3 Activity levels

**FIGURE 6:**
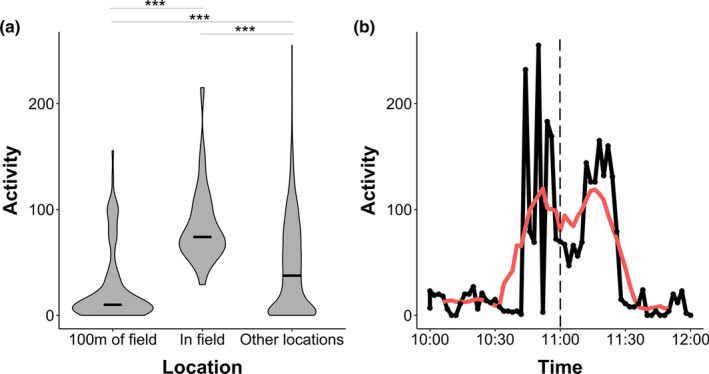
(a) Activity levels within 100 m of the field edge, in the fields and at all other GPS fixes. *** denotes a *p*‐value <.001. Black line indicates median values. (b) An example of an activity profile for the hour prior to and following a GPS fix in a field (at 11:00 on 06/01/14). Black line shows *x*‐axis accelerometer data averaged over two minutes. Red line shows a 10‐min moving average. Dashed line indicates the timing of a GPS fix in the crop field

Activity levels were significantly different between the field, within 100 m of the field and within the rest of the landscape (Figure 6a, Kruskal–Wallis rank‐sum test, *χ* ^2^ = 40.6, *df* = 2, *p* < .0001). Activity in the fields was significantly higher than at GPS fixes beyond 100 m of the field edge and also significantly higher than within 100 m of the field edge (Wilcox rank‐sum test, both *p* < .0001). Activity levels within 100 m of the field edge were significantly lower than for beyond 100 m of the field edge (Wilcox rank‐sum test, *p* < .0001).

These differences in activity in different areas are illustrated by an example of baboon activity levels prior to, during, and after a field visit on 06/01/14. GPS data indicate that the baboon was in the field at 11:00. At this time and in the minutes prior to and following this GPS fix, activity levels are significantly higher than at fixes outside of the fields (10:00 and 12:00). However, prior to this high activity, activity is very low, with an abrupt shift between the two (Figure 6b).

## 4 DISCUSSION

We applied a bio‐logging device collecting GPS and accelerometer data to a chacma baboon that foraged in commercial crop fields in South Africa to gain novel insights into its behavior without the need for direct observation. GPS data showed that ranging patterns changed through the year, with core home range forming three distinct areas, which did not overlap with the crop fields, suggesting that fields are perceived as high risk. The presence of the baboon in and around fields was temporally clustered at a small number of times across the year. As predicted, this generally overlapped with low plant primary productivity and likely low food availability and visits to the field and to the area within 100 m of the field edge were predominantly before 15:00. As expected, accelerometer data showed that activity was significantly higher within fields and significantly lower on the peripheries of fields relative to the rest of the ranging area, indicating that the baboon used a “sit‐and‐wait strategy,” engaging in lower activity behaviors on the field edge to assess risk before using high activity bursts to forage on crops. Together, these results provide novel insights into baboon crop‐foraging in commercial agriculture that can be used to inform effective crop protection strategies.

The baboon was recorded in crop fields on relatively few days during the study period (24 of 358 days). Though the GPS sampling frequency (one fix per hour) means that this is likely to be an underestimation of actual field visits, GPS fixes within 100 m of the field edge were also restricted to a small number of days, in close temporal proximity to those days when the baboon entered the fields. For the rest of the year, the baboon avoided the fields and their close surroundings, evidenced by the fact that the core home range does not overlap with the fields. Although the 95% home range does center on the fields, this is likely to be because water dictates the location of both baboons and fields, as water is needed to irrigate crops and also supports larger trees which baboons use as sleeping sites. Hence, baboon home ranges are likely to overlap with fields, but the fields themselves probably do not drive this pattern. The avoidance of the fields themselves and their close surroundings suggests that they are perceived as risky areas. Though risks may vary on a seasonal basis (crop guards are only present when crops are), these areas will have a higher risk of encountering humans than the surrounding landscape year‐round and have little vegetation cover. Combined with the fact that these fields are likely to have few beneficial uses for baboons when crops are not present, it is perhaps unsurprising that these areas are avoided. Spatial avoidance of areas with high levels of anthropogenic influence is a pattern seen in other mammalian taxa (e.g., Norum et al., 2015; van Doorn & O'Riain, 2020). Humans can create landscapes of fear (Laundré et al., 2001) by acting as an “apex super predator,” with greater effects on spatial and temporal patterns of animal activity than nonhuman predators (Clinchy et al., 2016). However, anthropogenic food sources can counteract this effect at certain times, attracting baboons to fields in spite of the risks posed and subsequent fear.

Field visits by the study baboon generally coincided with low NDVI. The exception to this was January 2014, when despite higher NDVI, field visits still occurred. It is likely that this was due to a lag in the availability of natural foods; despite high NDVI and therefore high primary productivity, fruits and seeds were not yet present, as there will be a delay between plants increasing primary productivity and fruit and seed production, after low NDVI in December. Furthermore, visits in January were all in the first half of the month (before the 14th of January), when NDVI would still be on the rise. Daily travel distance also increased as NDVI decreased, suggesting that the baboon was traveling further to forage for food when natural vegetation was lower, indicating a scarcity of food in the environment at times of lower NDVI. Though it is not possible to be certain that a lack of natural food drives crop‐foraging for all individuals in the group (as drivers of crop‐foraging are likely to vary depending on age and sex; Schweitzer et al., 2017; Strum, 2010), previous research looking at an entire group foraging on commercial farms in the same area, of all age‐sex categories, also found that crop‐foraging increased with lower NDVI (Findlay & Hill, In press). Therefore, along with previous research, these data support the hypothesis that baboons may be using crops as a “fallback food” (Hill, 2018; Marshall & Wrangham, 2007) in the study region.

Based on farmer reports and observations, baboons only enter fields when crops are present. Increased attraction to crop fields when crops are present has also been seen in other species; mountain gorillas were more likely to forage outside of Bwindi Impenetrable National Park in Uganda when palatable crops were present (Seiler & Robbins, 2016). Fields without crops are an unattractive habitat as they have little food or refuge and a high risk of potentially dangerous encounters with humans. Indeed, the study baboon avoided the crop fields for most of the year and core home range did not overlap with the fields. Unfortunately, it was not possible to collect reliable data on the timings of crop planting, ripening and harvesting in the fields in the study area. Interviews were conducted with farmers, but farmer plans were subject to significant change. However, highly temporally clumped visits to the fields and their peripheries strongly suggest that an important resource was attracting the baboon to the fields specifically at these times, most plausibly the presence of ripe crops. It is likely that a lack of crops also explains the lack of crop‐foraging in August, September, and November. However, because reliable crop data were unavailable, it is possible that other factors, such as shifts in ranging patterns, could also explain patterns of crop‐foraging and that crop‐foraging could be more opportunistic. Nevertheless, previous research on another group in the region showed an increase in the amount of crops removed from fields when NDVI values dropped below 0.32, despite crops being present prior to this (Findlay & Hill, In press). This suggests that it is seasonal changes, likely reflecting concomitant declines in natural food availability, that attract baboons to the fields rather than the presence of crops alone.

Visits to the field and to the area within 100 m of the field edge were predominantly before 15:00. This fits with previous research in the area, where greater crop‐foraging occurred in the morning than the afternoon (Findlay & Hill, In press). It was suggested that this was likely due to the close proximity of sleeping sites to the crop fields. However, the distribution of the sleeping sites for the baboon in this study (Figure 3a) suggests that this is unlikely to explain temporal patterns of crop‐foraging in this case. It has also been suggested that greater crop‐foraging in the morning may reflect a need to find food on waking (Priston, 2005). Further research is required to establish whether greater crop‐foraging in the morning is a general trend for chacma baboons in this part of South Africa.

Though accelerometers do not directly record behavior, heightened activity in the fields is likely due to running and provides further evidence for these areas being perceived as high‐risk environments by the study baboon. This heightened activity is likely to be in response to the crop guards employed to chase animals away from crops. Previous interviews with farmers in the area and behavioral observations support this conclusion (Findlay, 2016); baboons run into fields to collect crop items and retreat quickly to consume them away from fields. Baboons in Cape Town, South Africa, also used high activity forays to exploit anthropogenic food sources and their activity levels increased as the risk of deterrence from guards increased, providing evidence for a causal link between risk and activity levels (Fehlmann, O'Riain, Kerr‐Smith, et al., 2017). High activity in the fields also means that crop‐foraging is likely to be energetically costly. Discussion of the cost–benefit trade‐offs of crop‐foraging often focuses on the increased risk of injury and death for animals (e.g., Hill, 2018). However, it may also be important to consider the energetic costs of crop‐foraging when trying to deter animals from crop fields. Ensuring that costs of crop‐foraging outweigh benefits will underpin any effective crop protection strategy.

The baboon showed low activity within 100 m of the field edge. This suggests that the baboon spent time waiting near to anthropogenic food sources engaging in low activity behavior to assess risks and wait for an opportunity to forage when the chance of detection and chasing from field guards was lowest: a “sit‐and‐wait” strategy. Previous observations of another group in the region suggest that baboons spend a significant amount of time near the field edge, entering fields for relatively short periods to engage in crop‐foraging (Findlay, 2016). However, it was not possible in that study to establish what baboons were doing on the field peripheries through direct observation, though it was observed that baboons often entered fields at the opposite side of the field to guards (Findlay, 2016), a “strategy” that has also been observed in yellow baboons in Kenya (*Papio cynacephalus*; Maples et al., 1976). Together, this supports the idea that they are using this time on the edge of fields to assess risk and choose an appropriate time and location to crop forage. This pattern is also mirrored in the behavior of urban foraging baboons in South Africa; collared baboons foraging in anthropogenic areas in and around Cape Town spent the majority of their time on the urban edge at relatively low activity, spending very little time in the urban areas themselves (Fehlmann, O'Riain, Kerr‐Smith, et al., 2017).

Our results show consistency with the “sit‐and‐wait” strategy used by urban foraging chacma baboons in Cape Town, South Africa, with baboons waiting near to anthropogenic feeding opportunities and then using high activity behaviors to access urban food sources and crops (Fehlmann, O'Riain, Kerr‐Smith, et al., 2017). This is despite the significant differences between the two contexts; urban foraging baboons navigated a much more heterogeneous environment compared with the homogeneity of crop fields and urban food sources are spaced out over a greater area. However, the behavioral strategies were very similar. This consistency in response could be positive for addressing human–wildlife conflict, as management strategies for crop‐foraging may be applicable to urban areas and vice versa.

These insights into baboon behavior can inform management of human–primate conflict. Increasing natural food availability in the dry season by planting species that provide fruits and seeds at this time could reduce the prevalence of baboon crop‐foraging as a fallback strategy. The vast majority of crop‐foraging also appears to occur before 15:00. At present, guards are hired from dawn to dusk, but focusing guarding efforts on the morning and early afternoon could increase guard effectiveness, though monitoring would be required to ensure that this does not result in temporal displacement of crop‐foraging. The collared baboon also appeared to perceive the crop fields as risky areas, but not areas just beyond the field boundary, illustrated by the low activity levels within 100 m of the field edge. Crop guards only chase baboons when they are in the fields (Findlay & Hill, In press). Chasing baboons both when close to the fields and when in the fields could prevent baboons spending time in close proximity to the fields engaged in low activity behaviors. In addition, buffer zones around fields without vegetation for refuge could help to discourage baboons from spending time at the field periphery engaged in low activity behaviors. Buffers of cleared land have not been systematically tested as a crop protection strategy (Junker et al., 2017), possibly as clearing land often reduces the size of arable land or protected areas. However, in our study context the habitat surrounding fields is not of great conservation value and water availability rather than available space determines the area of land cultivated.

This data set provides a useful platform for future work. Larger sample sizes across multiple groups would help to establish whether the relationship between natural food availability and crop‐foraging holds true across multiple groups and multiple seasons. It is also important to recognize that there may be significant age and sex stratification in crop‐foraging patterns (Schweitzer et al., 2017; Strum, 2010) so the data collected from the single study baboon, an adult female, may not be wholly representative of the entire group. Larger sample sizes, along with GPS data at a higher temporal and spatial resolution, could shed light on other baboon crop‐foraging strategies, including heterogeneity within groups. Ground‐based measures of food and crop availability would strengthen links between ecology, crop availability, and crop‐foraging. Triaxial accelerometers can also give insights beyond simple measures of activity, with machine learning capable of matching accelerometer profiles to specific behaviors, giving a greater understanding of what animals are doing without observer presence (Fehlmann, O'Riain, Hopkins, et al., 2017). In particular, it would be useful to understand baboon behavior on the field periphery at greater resolution; grooming and vigilance behavior both have similar low activity yet reflect differing perceived risks in these areas.

This research not only provides insights into crop‐foraging by baboons that can be used to reduce human–wildlife conflict, but also provides a basis for further, more detailed work using bio‐logging technology. Furthermore, any insights gained into chacma baboons in this context may apply to crop‐foraging by other primates, which could be useful in mitigating conflict between humans and other species threatened with extinction.

## CONFLICT OF INTEREST

None to declare.

## AUTHOR CONTRIBUTION

**Ben John Walton:** Conceptualization (equal); Data curation (lead); Formal analysis (lead); Methodology (equal); Visualization (lead); Writing‐original draft (lead); Writing‐review & editing (equal). **Leah J Findlay:** Conceptualization (equal); Investigation (lead); Methodology (equal); Writing‐review & editing (equal). **Russell A Hill:** Conceptualization (equal); Funding acquisition (lead); Supervision (lead); Writing‐review & editing (equal).

## Data Availability

All data used in this publication are available from the Dryad data repository, https://doi.org/10.5061/dryad.zw3r22870
